# Selenocysteine insertion sequence binding protein 2 (Sbp2) in the sex-specific regulation of selenoprotein gene expression in mouse pancreatic islets

**DOI:** 10.1038/s41598-020-75595-4

**Published:** 2020-10-29

**Authors:** B. Chellan, L. Zhao, M. Landeche, C. M. Carmean, A. M. Dumitrescu, R. M. Sargis

**Affiliations:** 1 Division of Endocrinology, Diabetes, and Metabolism, Department of Medicine, University of Illinois at Chicago, 835 S. Wolcott, Suite E625; M/C 640, Chicago, IL 60612 USA; 2 Section of Endocrinology, Diabetes, and Metabolism, Department of Medicine, University of Chicago, Chicago, IL USA; 3 ChicAgo Center for Health and EnvironmenT (CACHET), Chicago, IL USA

**Keywords:** Cell biology, RNA metabolism, Transcription, Diabetes

## Abstract

Selenoproteins are a group of selenocysteine-containing proteins with major roles in cellular antioxidant defense and thyroid hormone metabolism. Selenoprotein expression is determined by hierarchical mechanisms that result in tissue-specific levels. Current data inadequately explain the abundance of various selenoproteins under normal and pathological conditions, including in pancreatic β-cells. Selenocysteine insertion sequence binding protein 2 (SBP2) is a critical protein in selenoprotein translation that also plays an essential role in stabilizing selenoprotein transcripts by antagonizing nonsense-mediated decay (NMD). Importantly, dysfunctional SBP2 is associated with endocrine disorders in humans. Here we describe the impact of induced *Sbp2* deficiency in pancreatic β-cells on selenoprotein transcript profiles in the pancreatic islets of C57BL/6J mice. Sex differences were noted in control mice, in which female islets showed 5 selenoproteins decreased and one increased versus male islets. Induced *Sbp2* deficiency in pancreatic β-cells altered expression of only 3 selenoprotein transcripts in male islets, whereas 14 transcripts were reduced in female islets. In all cases, decreased transcription was observed in genes known to be regulated by NMD. The differential impact of *Sbp2* deletion on selenoprotein transcription between sexes suggests sex-specific hierarchical mechanisms of selenoprotein expression that may influence islet biology and consequentially metabolic disease risk.

## Introduction

The pancreatic islets of Langerhans proteome plays a central role in the homeostatic regulation of glucose metabolism. A subset of this proteome consists of selenoproteins, a family of proteins in which selenium is incorporated into the protein structure as the unique amino acid selenocysteine (Sec). These selenoproteins comprise a group of functionally diverse molecules found in all major life forms. There are 24 and 25 known genes encoding selenoproteins in mouse and humans, respectively^1^. These proteins perform varied functions related to thyroid hormone metabolism, redox biology, and immune response among others. Despite the importance of these proteins in multiple aspects of cellular biology, the role of selenoproteins has not been fully elucidated in islet or β-cell biology. This is relevant as cellular antioxidant functions of selenoproteins are a critical component of antioxidant defense^2,3^, and oxidative stress is thought to play a key role in the β-cell dysfunction that contributes to the pathogenesis of diabetes^4^.

During selenoprotein translation, an in-frame UGA stop codon in the selenoprotein mRNA is recoded as the selenium-containing amino acid Sec, which is incorporated into the protein structure by a complex multicomponent machinery. Key to this process are the Sec-insertion sequence (SECIS) element in the 3′-untranslated region (UTR), a Sec-specific elongation factor (EFsec), and the SECIS-binding protein-2 (SBP2)^2,5–7^. Binding of SBP2 to SECIS elements on selenoprotein mRNA and the coordinated events that follow are essential for protecting selenoprotein mRNA from premature degradation mediated by nonsense-mediated decay (NMD), a native post-transcriptional process that orchestrates the degradation of premature stop codon-containing mRNA transcripts^8,9^. Thus, in addition to its role in selenoprotein translation, SBP2 is essential for the upstream stabilization of selenoprotein mRNA.

Because of their essential roles in regulating cellular oxidative homeostasis, it is likely that disruptions in selenoprotein synthesis will lead to cellular dysfunction and consequential adverse health effects. Those precise effects, however, are likely tissue-specific and dependent upon the precise functions of the complement of selenoproteins expressed in that specific tissue. For example, deficiencies of the micronutrient selenium, a limiting factor for selenoprotein synthesis, is implicated in the development of human cardiomyopathy and other adverse health effects^7,10,11^; moreover, it has also been suggested to impact insulin secretion and islet function in rodents while contributing to insulin resistance in humans^12,13^. Further evidence that disruptions in selenoprotein synthesis cause human disease comes from evidence of humans with dysfunctional SBP2, which is associated with thyroid dysfunction^14^ and neurocognitive deficits^15^ as well as azoospermia, muscular dystrophy, photosensitivity, and immune dysfunction^16^.

Considerable variation in selenium demand and selenoprotein expression has been reported across different tissues. Furthermore, plasma selenium status affects tissue levels of selenoproteins differentially; while some selenoproteins are affected by selenium deprivation, others are not^3,17,18^. Additionally, the complex nature and diversity of selenoprotein function complicates assignment of adverse health effects to specific selenoproteins^17^. Thus, understanding the precise complement of selenoproteins in a specific tissue and the regulation of their expression levels is critical for understanding how these essential proteins regulate function at the level of specific tissues. A critical role of SBP2 in selenoprotein biosynthesis is determined by the differential binding affinity to various SECIS elements of selenoprotein mRNAs^6^, which influences the variable tissue expression of selenoproteins. Consequently, β-cell-specific deletion of *Sbp2* may illuminate vital information about the selenoprotein expression hierarchy that may be essential for pancreatic β-cell function and glucose homeostasis. Previously, mouse models conditionally deficient in *Sbp2* gene expression have been proposed as models to study the tissue-specific function of individual selenoproteins^19^, here we present the selenoprotein gene expression profiles in pancreatic islets from mice we developed that have an inducible β-cell-specific *Sbp2* gene deletion and discuss the sex-specific differential gene expression of selenoproteins in murine islets.

## Methods

### Development of conditional knockout mice and induction of β-cell-specific *Sbp2* deficiency

C57BL/6J mice with β-cell-specific, tamoxifen-inducible deletion of the *Sbp2* gene (*Sbp2* βCKO mice) were generated by cross-breeding mice homozygous for the floxed *Sbp2* allele (*Sbp2* flox/flox (*Sbp2 fl/fl*)) with *Sbp2 fl/fl* mice that are also hemizygous for the Mip-Cre-ERT transgene, expressing Cre recombinase driven by the mouse Ins-1 promoter (Mip-Cre-ERT/+; *Sbp2 fl/fl*). *Sbp2 fl/fl* mice were generated by cross-breeding heterozygous *Sbp2* floxed mice (*Sbp2*+*/fl*) previously reported by our team^20^. Mip-Cre-ERT mice were procured from Jackson Laboratories (Bar Harbor, ME). Male and female littermate mice at 15 weeks of age were used in the study. *Sbp2* deficiency was induced by intraperitoneal administration of 3 mg tamoxifen dissolved in corn oil for 5 consecutive days, after which mice were allowed to recover for 3.5 weeks prior to analysis of *Sbp2* gene and protein deletion as well as selenoprotein gene expression in isolated pancreatic islets of Langerhans. Mice were group housed under 14-h/10-h light/dark cycles at 22.2 °C ± 1.1 °C with ad libitum access to a selenium-replete chow diet (Teklad 7912) and water. The recommended selenium requirement for adult laboratory mice is 0.15 mg/kg of diet^21^; Teklad 7912 diet contains 0.16 mg/kg of selenium. All animal protocols were approved by the Institutional Care and Use Committee at the University of Illinois at Chicago (Protocols ACC 16-211 and ACC 19-198), and all experiments were conducted in accordance with the National Institutes of Health Guide for the Care of Laboratory Animals.

### Isolation of pancreatic islets

Mouse pancreatic islets were isolated using methods previously described^22,23^. Briefly, collagenase P (Roche) at a final concentration of 0.375 mg/ml in HBSS buffer containing HEPES (10 mM) at pH 7.2 was perfused through the pancreas by cannulation of the common bile duct starting at the gall bladder while the ampulla remained clamped preventing perfusate flow into the duodenum; pancreata were digested at 37 °C for 14 min. The digested pancreas was then gently dispersed in cold wash buffer (HBSS-HEPES pH 7.2 containing 0.01% BSA) and washed thrice before handpicking the dispersed islets under stereomicroscopy.

### RNA isolation and qPCR

Total islet RNA was isolated using the EZNA Total RNA Kit2 (Omega Biotech). Islets from each mouse were homogenized by passage through a 23-gauge needle, and total RNA was isolated according to the manufacturer’s instructions. RNA was further purified by digesting genomic DNA using DNAse 1 (Invitrogen). A total of 0.4 μg RNA was reverse transcribed using qScript cDNA Super Mix (Quanta Bio). The abundance of mRNA was measured by real-time quantitative PCR (qPCR) using the Bio-Rad CFX Connect real-time system and SYBR green detector dye. Primer sequences for genes analyzed in the study are listed in Table 1. All primers were validated and found to have optimal efficiency (E = 90–110%, R^2^ =  > 0.98, slope = − 3.58 to − 3.1). Gene expression was normalized to the expression of the house-keeping gene *Hprt*.

**Table 1:** Primer sequences for quantitative real-time PCR (qPCR).

Gene	Forward primer	Reverse primer
*Sephs2*	CCCACCAATGGCTGGATAAT	GCAGCAGTCCTGTTTAGAGTAG
*Selenop*	ATCAACCAGCTCCTGTGTAAG	GCAGACCCTGACTTCTCAAATA
*Gpx1*	CGACATCGAACCTGACATAGAA	CAGAGTGCAGCCAGTAATCA
*Gpx2*	CCTACCGGCCATTTCCTTTA	GACATCTCCCAGAAGGGTTTAG
*Gpx3*	CCCTTAGTGCATTCAGGCTTAG	GATACCAGTGGACAGAGTGAGA
*Gpx4*	CCGATATGCTGAGTGTGGTTTA	GGCTGCAAACTCCTTGATTTC
*Gpx6*	AACCAAACCAGTACCCAGTAAG	GCATTAGTAGGGACACAGGAAG
*Txnrd1*	ACAGCGAGGAGACCATAGA	CCACGGTCTCTAAGCCAATAG
*Txnrd2*	CATCTTCTGGCTGAAGGAGTC	ACAGTGGTATCCAGTCCAATTC
*Txnrd3*	GAAGGACCGGAAACAGTAGAA	CGATCTTCTCCAGCCCTATTT
*Msrb1*	CTCGAAGTACGCACACTCATC	CTTGCCACAGGACACCTTTA
*Dio1*	CAGGATCTGCTACAAGGGTAAAG	GTGTCTAGGTGGAGTGCAAAG
*Dio2*	TCCTACAGTCACAATGCTACAC	GGTTAGTGTACCTGCCTCTTAC
*Dio3*	CTCGAAACAGCGCCTAAAGTA	GAAGTCCATCCCTTACCATGTC
*Selenof*	GAAGTAGCACCACAGTCCATAA	GGTGAGAGCAACAAGTGTAGAA
*Selenok*	GTGCAGGAGAAGATGGTGTATG	TGCGTATCAGTCAGGCTAGA
*Selenos*	CTTTGCGAGGAGGTGGTTAT	GTCAGAGCGACACTAACAAGAG
*Selenom*	GTCACCGAGGACATTCAACT	GTTCCTGGTAATTTCGGCTTAAC
*Selenon*	TGTTGACCTGATGACCCAAG	CTAGACAGTGCTGGCAATAAGA
*Selenot*	GAAGTTATGAGCTGAGGGAAGG	CAGTGTGTGTCCAGTAGTCAAG
*Selenoh*	CAGTCCCTGGAGATGTTGAAG	TCACTTCTGGTGAGGAAGAAAC
*Selenoi*	GGAGTGTTCTGCCGTTACTT	GAGGAACCGCCACAATTAGA
*Selenoo*	CACTATCGACTATGGACCCTTTG	GGGCTGCTTACTGTATGTGTAG
*Selenov*	GCTATGGCCTTCGGTACATTAT	CTGTAACCTGGGTAGCTCTTTC
*Selenow*	TCACCGGGTTCTTTGAAGTG	CCGGAACTTGCTCTCTGTATC
*Hprt*	GGCCAGACTTTGTTGGATTTG	CGCTCATCTTAGGCTTTGTATTTG
*Sbp2*	AAGACCGCATGTACCAGAAG	CTCACAGTTGGGAGAGATGATG
*Lrp1*	ACTATGGATGCCCCTAAAACTTG	GCAATCTCTTTCACCGTCACA
*Lrp2*	CAACGGAGCCTGCTATAACAC	TTTTTGCGAACACAGCGTAGT
*Lrp8*	TCCTGCCGAGAAGTTAAGCTG	AAGAACGCAAGTCCCATCCC

### SDS-PAGE and immunoblotting

Tissue proteins were extracted in RIPA buffer supplemented with complete Mini-Protease Cocktail (ROCHE). Primary antibodies against Sbp2, Gpx3, Dio1, and Txnrd2 (all from Proteintech) as well as Selenop (Santa Cruz) were used to quantify select selenoprotein expression by immunoblotting after separation by SDS-PAGE; β-actin (Cell Signaling) was used as the internal protein loading control. Clarity ECL (Bio-Rad) was used for chemiluminescent detection of protein bands with band intensity quantified using Image J software.

### Statistics

All data are presented as means ± standard deviations (SD). Statistical significance was tested using two-tailed, unpaired Student’s *t* tests to compare control and *Sbp2* knockout groups. All statistical analyses and image construction were performed using GraphPad Prism and Adobe Illustrator; *p* < 0.05 was considered statistically significant.

## Results

### Mice with tamoxifen-induced conditional Sbp2 deficiency in pancreatic β-cells

**Figure1:**
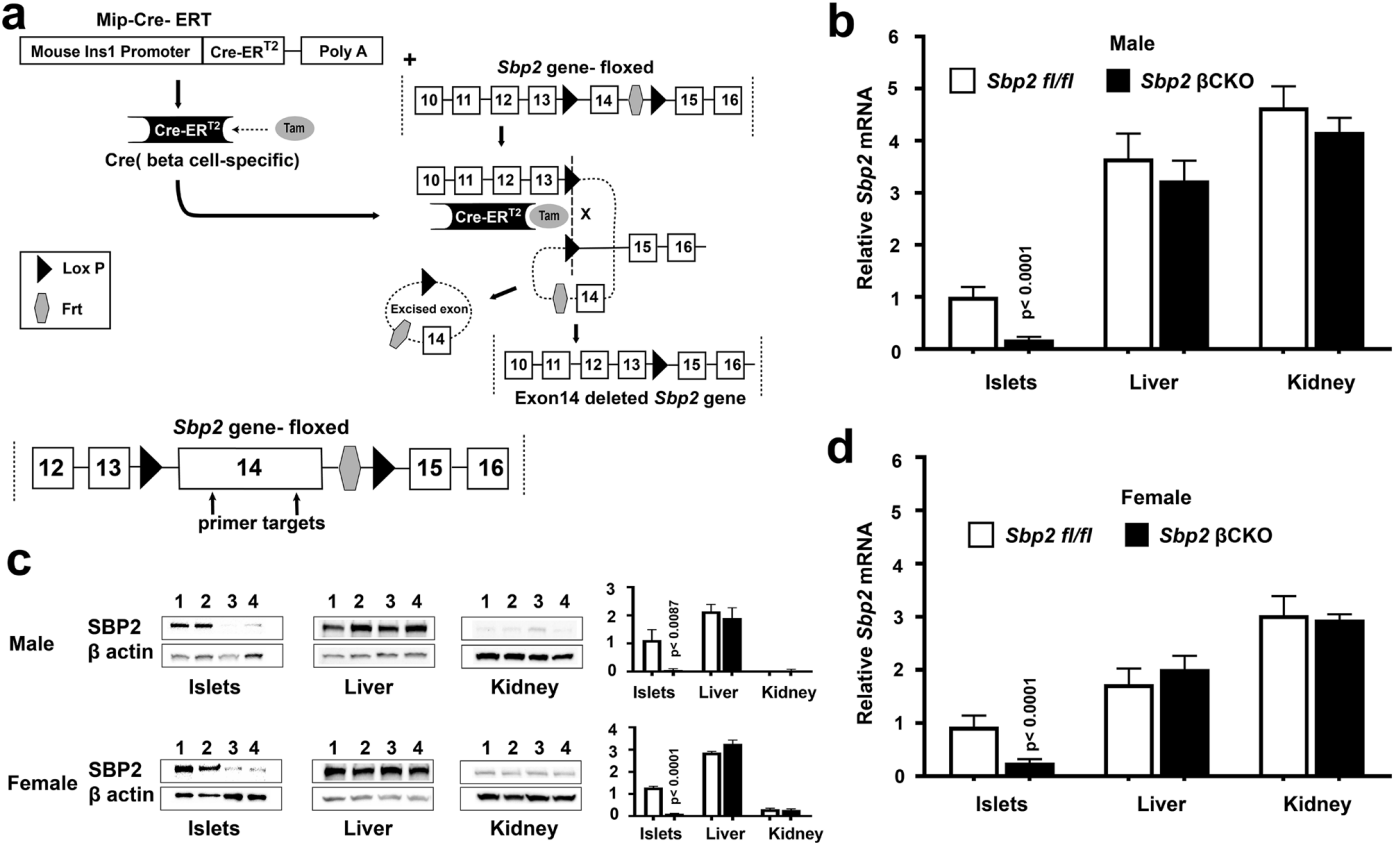
*Sbp2* gene targeting and characterization in *Sbp2* βCKO mice. (**a**) Graphical representation of genetic combinations used to breed *Sbp2* βCKO mice. The mouse *Sbp2* gene contains 17 exons. *Sbp2* βCKO mice are hemizygous for the Mip-Cre-ERT transgene and homozygous for the floxed *Sbp2* gene (exon 14 flanked by *LoxP* sites). Specific expression of Cre-recombinase in β-cells is induced upon tamoxifen binding, which triggers LoxP recombination and excision of exon 14. The *Frt* site that remained after Flp-induced excision of the *Neo*^*R*^ cassette during the making of *Sbp2* floxed mice is also excised along with exon 14 upon Cre-Lox recombination in the *Sbp2* βCKO mice as shown. *Sbp*2 qPCR primer-binding locations within exon 14 are also shown. Male (**b**) and female (**d**) *Sbp2* βCKO mouse islets, liver, and kidney were analyzed for *Sbp2* mRNA expression by qPCR and compared to control *Sbp2 fl/fl* mice lacking the Mip-Cre-ERT transgene (*Sbp2 fl/fl*) but also treated with tamoxifen. (**c**) Representative immunoblots showing Sbp2 protein expression for islets, liver, and kidney in *Sbp2* βCKO and *Sbp2 fl/fl* controls in both male (top panels) and female (bottom panels) mice; lanes 1 and 2 are from control mice (*Sbp2 fl/fl*), and lanes 3 and 4 are from *Sbp2* βCKO mice; β-actin was used as the protein loading control. Corresponding quantitation of protein levels was normalized to β-actin expression in arbitrary units and is shown at the right. Between group differences were analyzed by unpaired, two-tailed Student’s *t* test. N = 6 mice per group; *p* < 0.05 was considered statistically significant with individual* p* values as shown.

Constitutive *Sbp2* gene deletion is embryonically lethal in mice; therefore, we generated transgenic mice (*Sbp2* βCKO) with tamoxifen-inducible *Sbp2* gene deletion specifically targeting pancreatic β-cells. This approach avoids the potential effect of congenital absence of *Sbp2* on β-cell development. *Sbp2* βCKO mice (Mip-Cre-ERT/+; *Sbp2 fl/fl*) harbor the mouse insulin promoter (Mip)-driven Cre-ER transgene that expresses the fusion protein Cre recombinase–estrogen receptor (ER). Binding of tamoxifen to the ER translocates the Cre recombinase into the nucleus^24^ where the homologous recombination of the LoxP sites flanking exon 14 of the *Sbp2* gene results in its deletion (Fig. 1a). Exon 14 was targeted for deletion as it encodes part of the C terminus functional domains that are highly conserved across species and required for SECIS binding, ribosome binding, and Sec incorporation. It is present in all known isoforms encoded by this locus, and when deleted, the resultant transcript between exons 13 and 15 is out of frame, ensuring a nonfunctional transcript and a null allele. *Sbp2*-floxed mice were originally developed by our team^20^ and *Sbp2* βCKO were then generated by crossing Mip Cre-ERT mice with *Sbp2* floxed mice as described. Tamoxifen was administered at 15 weeks of age to both *Sbp2* βCKO and littermate *Sbp2 fl/fl* control mice lacking Cre. Profiling of mRNA expression of the mouse islets showed 75% and 79% *Sbp2* gene deletion in female and male *Sbp2* βCKO mice, respectively (Fig. 1b,d). SBP2 protein was similarly decreased in both male and female *Sbp2* βCKO mice (Fig. 1c). To ascertain whether β-cell-specific Cre expression had nonspecific effects in other tissues, we assessed *Sbp2* gene expression in liver and kidney and found that there was no detectable deletion of *Sbp2* in these tissues (Fig. 1b,d). Importantly, β-cells constitute approximately 60–80% of the cellular composition of murine islets, with an additional 15–20% being α-cells and the rest of the islet composed of δ- and PP-cells^25^. With targeted deletion of *Sbp2* in β-cells, the extent of *Sbp2* deletion reflects the β-cell composition of the pancreatic islets at both the mRNA and protein level in both male and female mice (Fig. 1). Of note, recombination was largely uniform in all the mice with none yielding less than 65% *Sbp2* gene deletion (data not shown). Importantly, tamoxifen administration and gene deletion did not induce overt adverse health effects in the mice. Taken together our results show that our model successfully induces *Sbp2* deficiency in pancreatic β-cells.

### Transcriptional analysis of selenoproteome in male and female murine islets

**Figure 2:**
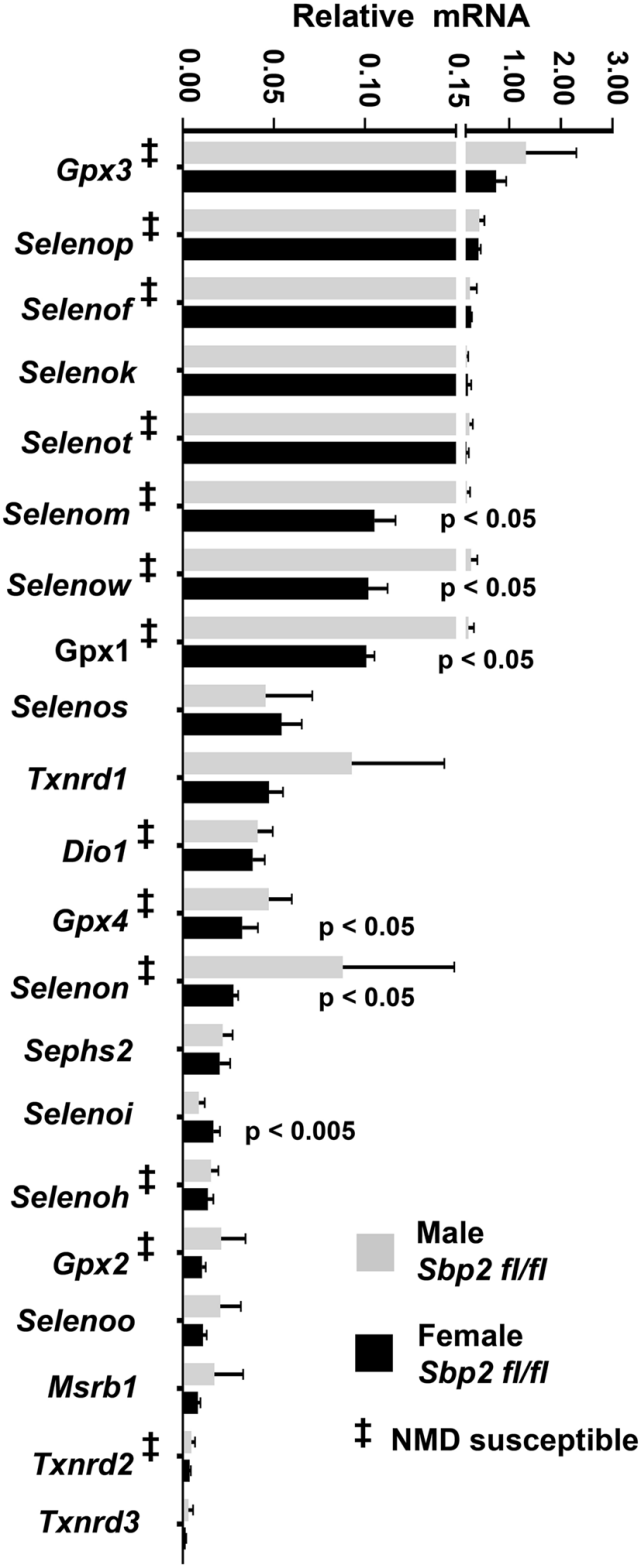
Selenoprotein gene expression analysis in *Sbp2 fl/fl* control male and female mouse islets. Quantitative real time-PCR analysis (qPCR) of selenoprotein mRNA abundance in male and female control mice (*Sbp2 fl/fl*) is shown. Individual selenoprotein mRNA expression is displayed compared relative to the expression of *Gpx3*, which was the most abundantly expressed selenoprotein mRNA in both male and female islets. Expression was normalized to the housekeeping gene *Hprt*. Genes susceptible to NMD are marked with the following symbol: ^‡^. These are selenoprotein pre-mRNAs with a UGA stop codon at least 50–55 nucleotides upstream of an intron. Between group differences were analyzed by unpaired, two-tailed Student’s *t* test. N = 6 mice per group; *p* < 0.05 was considered statistically significant with individual *p* values as shown.

To characterize the islet selenoprotein profile, we performed transcriptional analyses of all 24 mouse selenoprotein genes in the pancreatic islets of *Sbp2* βCKO mice and their control littermates (*Sbp2 fl/fl*). Sexual dimorphism in selenoprotein expression has been reported in murine tissues and plasma that may explain several sex-specific metabolic functions in mouse^29,30^. However, data is lacking regarding such sex specific selenoprotein expression in the pancreatic islets. We found that mRNA expression of *Selenom, Selenow, Gpx1, Gpx4*, and *Selenon* (all NMD-susceptible) were decreased while *Selenoi,* a non-NMD-susceptible selenoprotein was significantly increased in the female control mouse (*Sbp2 fl/fl*) islets compared to their male counterparts (Fig. 2).

**Figure 3:**
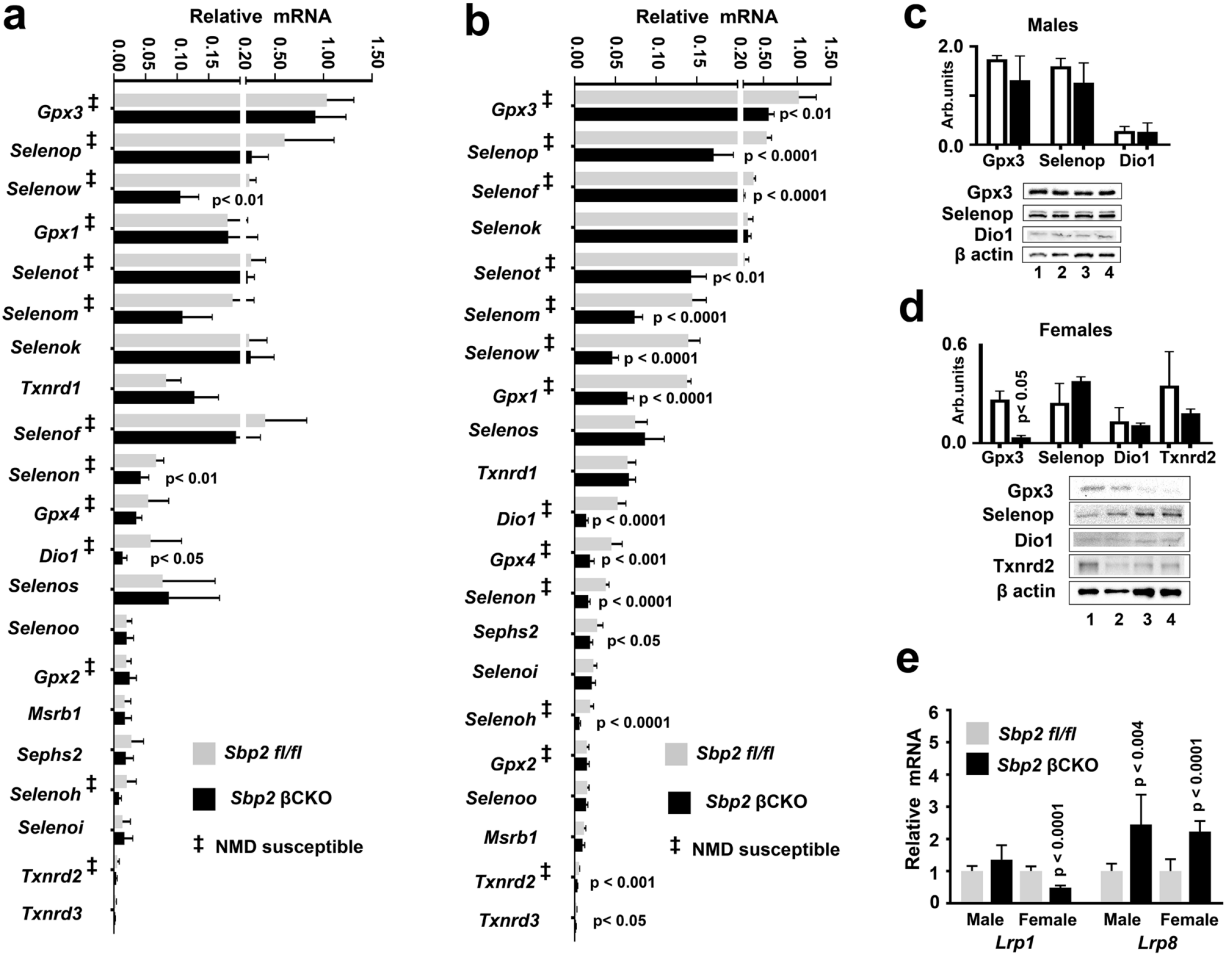
Selenoprotein gene expression analysis in *Sbp2*-deficient male and female mouse islets. Quantitative real time-PCR analysis (qPCR) of selenoprotein mRNA in male (**a**) and female (**b**) *Sbp2* βCKO islets compared with that of control mouse islets (*Sbp2 fl/fl*). Individual selenoprotein mRNA expression is displayed compared relative to the expression of *Gpx3*. Expression was normalized to the housekeeping gene *Hprt*. Genes susceptible to NMD are marked with the following symbol: ^‡^. Representative immunoblots showing Gpx3, Selenop, Dio1, and Txnrd2 protein expression in male (**c**) or female (**d**) islets of *Sbp2* βCKO and control (*Sbp2 fl/fl*) mice are shown; lanes 1 and 2 are representative islets from control mice (*Sbp2 fl/fl*), and lanes 3 and 4 are representative islets from *Sbp2* βCKO islets; β-actin was used as the protein loading control. Corresponding quantification expressed in arbitrary units is shown at the right normalized to β-actin expression. (See also Supplementary Information 1.) Gene expression of the Selenop receptors *Lrp*1 and *Lrp*8 in male and female *Sbp2* βCKO islets compared with that of control mouse islets (*Sbp2 fl/fl*) is shown in (**e**). Between group differences were analyzed by unpaired, two-tailed Student’s *t* test. N = 6 mice per group; *p* < 0.05 was considered statistically significant with individual *p* values as shown.

Importantly, SBP2 induces recoding of a UGA stop codon in selenoprotein pre-mRNA^5^, and SBP2 is essential for prevention of nonsense-mediated decay (NMD) of these transcripts^8,9^. NMD in mammals is activated when a pre-mRNA contains a stop codon (UGA) at least 50–55 nucleotides upstream of an intron^8,9^; 14 of the 24 mouse selenoprotein pre-mRNAs are known to be susceptible to NMD^9^. In male *Sbp2* βCKO mice with greater than 70% deletion of *Sbp2* (Fig. 3a), the expectation was to see similar reductions in mRNA levels of the full complement of NMD-susceptible selenoproteins. Surprisingly, islet-selenoprotein mRNA expression was largely unchanged by *Sbp2* deletion with only marginal reductions in the expression of *Selenow*, *Selenon*, and *Dio1*, all of which are susceptible to NMD (Fig. 3a). In contrast to male islets, however, 14 of the 24 selenoproteins were decreased in islets from female *Sbp2* βCKO mice; all of which are NMD-susceptible (Fig. 3b). *Gpx3* and *Selenop* were the most highly expressed selenoproteins in both male and female islets, while *Txnrd2* and *Txnrd3* were the least expressed selenoproteins. *Dio2*, *Dio3*, and *Selenov* expression were not detected in islets from either male or female mice.

Protein expression of major selenoproteins (Fig. 3c,d), however, did not strictly reflect mRNA abundance. There were also notable sex differences. In islets from male mice, *Sbp2* deletion did not affect Gpx3 mRNA or protein expression, however, in islets from female mice, *Sbp2* deletion reduced *Gpx3* at both the gene and protein levels. This suggests possible post-translational mechanisms influencing protein expression that are sex-specific, as well as unknown sex-specific NMD control mechanisms that may be operative. Potential mechanisms for these sex specific differences in selenoprotein gene expression levels upon *Sbp2* deletion are not clearly understood.

Lrp1, Lrp2 and Lrp8 are known receptors for Selenop^31,32^, the major selenium transporter protein. Expression of *Lrp8* mRNA was previously shown to be strongly upregulated in Selenop*-*deficient mouse tissues, suggesting one potential compensatory mechanism to ameliorate the selenium deficiency in those tissues^31^. Similar to these studies, *Lrp8* was highly expressed in both male and female *Sbp2* βCKO mice islets compared to *Sbp2 fl/fl* controls (Fig. 3e). In contrast, no changes in *Lrp1* mRNA levels in male *Sbp2* βCKO mice islets were observed; however, female *Sbp2* βCKO mice islets showed reduced *Lrp1* expression relative to *Sbp2 fl/fl* controls (Fig. 3e). *Lrp2* mRNA was not expressed in mouse islets. Taken together our data indicate that *Sbp2* deletion in pancreatic β-cells does not broadly impact selenoprotein mRNA levels in male islets, while many female selenoprotein transcripts are significantly decreased, indicating sexually dimorphic effects of *Sbp2* deletion in β-cells.

## Discussion

Selenium and several selenoproteins are known to play diverse roles in the health and function of pancreatic islets^12,13,33,34^. Understanding the functions of the selenoprotein complement on pancreatic β-cell and islet function is essential for appreciating the importance of this family of proteins for metabolic health as well as their potential role in the pathogenesis of diabetes. Selenoproteins are expressed hierarchically in a tissue-specific pattern that is often based upon the availability of selenium and other physiological factors^26^. Moreover, some tissues preferentially retain selenium relatively independent of systemic selenium availability^26,27^. For example, selenium levels in endocrine tissues such as the rat pituitary and thyroid remain relatively unchanged in the face of dietary selenium deficiency in order to preserve essential selenoproteins^28^. The unique tissue-specific expression of selenoproteins suggests that protein and mRNA expression profiles across the selenoproteome may shed light on tissue-specific selenium needs and the precise selenoprotein expression hierarchy that regulates health and mediates disease risk. Importantly, however, these data are generally lacking.

Pancreatic islets of Langerhans secrete several hormones essential for metabolic homeostasis, and disruptions in this critical endocrine organ promote the development of metabolic disease. SBP2 expression is critical for mammalian selenoprotein expression; however, little is known about the selenoprotein expression hierarchy in β-cells and the role of SBP2 in this critical cell population. We found a 70–80% decrease in *Sbp2* gene expression in mouse islets in our model of β-cell-specific *Sbp2* deficiency upon tamoxifen induction. Of a potential 24 selenoproteins expressed in murine tissue, only 21 were expressed in isolated pancreatic islets; *Dio2*, *Dio3*, and *Selenov* were not expressed. Selenoprotein mRNA profiles have been described in SBP2-deficient mouse liver^19^. Seeher et al. reported results from a mouse with liver-specific *Sbp2* deletion in which *Sbp2* was deleted embryonically by albumin-driven Cre expression. While selenoproteins such as *Dio1*,* Gpx1*,* Selenop*,* Selenow*,* Selenoh*,* SephS2*, and* Selenos* were significantly downregulated in both male and female Sbp2-deficient mouse livers, others showed sexual dimorphism with respect to selenoprotein expression changes, including *Gpx4*,* Txnrd1*,* Selenot*,* Msrb1*,* Selenon*,* Selenof*,* Selenom*,* Txnrd1*,* Selenoi*,* Selenok*, and* Selenoo*^19^.

In contrast, our studies aimed to induce *Sbp2* deficiency during adulthood selectively in pancreatic β-cells by tamoxifen-mediated Cre induction. This approach has the advantage of bypassing effects on β-cell development and potential secondary effects mediated by life-long alterations in β-cell function. While only *Dio1*, *Selenom*, and *Selenow* were down regulated in male *Sbp2* βCKO islets, 14 transcripts were downregulated in female islets: *Gpx3, Selenop, Selenof, Selenot, Selenom, Selenow, Gpx1, Dio1, Gpx4, Selenon, Sephs2, Selenoh, Txnrd2, and Txnrd3*. Additionally, compared to their male littermates, islets from control female mice (*Sbp2 fl/fl*) exhibited reduced expression of 5 NMD-susceptible selenoproteins, while expression of the non-NMD-susceptible selenoprotein *Selenoi* was increased. Less clear, however, are the mechanisms responsible for the marked sex-specificity of our findings. It is known that NMD mechanisms vary in potency in a tissue-specific manner^35^; however, whether sex hormones and/or sex steroid receptors play a role in this process is not understood.

Glutathione peroxidases include Gpx1, Gpx2, Gpx3, Gpx4, and Gpx6; these are the best characterized selenoproteins with known roles in islet biology^5,36^. Global overexpression of Gpx is known to protect pancreatic β-cells from oxidative stress and improve insulin sensitivity^36,37^. In our studies, *Gpx3* was the most abundant selenoprotein mRNA expressed in islets, while *Gpx2* and *Gpx4* were comparatively very lowly expressed. Gpx3 mRNA and protein expression remained unchanged in male *Sbp2* βCKO mice; however, both mRNA and the protein levels were decreased in female *Sbp2* βCKO mice. Several mechanisms may explain the preservation of *Gpx3* in islets from male mice. In thyrocytes, GPX3 is known to selectively accumulate extracellularly in the colloid of the thyroid gland, where it is effectively isolated and shielded from circulation; this protects the protein from degradation and renders it resistant to changes in selenium status^26^. Such hierarchical mechanisms may operate in male islets as well. For example, in rats fed a selenium-deficient diets for several generations, GPx activities were found decreased to undetectable levels in several tissues, and male rats were found infertile compared to females; however, levels of selenium in the testis, brain, and endocrine tissue remained unchanged^26–28^.

Selenop (Sepp or Selenoprotein P) may play a role in regulating pancreatic β-cell function while also modulating insulin sensitivity in type 2 diabetes^38^. Selenop serves primarily as a selenium transport protein responsible for roughly 50% of plasma selenium^39^. Selenop is unique in that it carries more than one selenocysteine (Sec) residue in its structure; the number of which varies across species. For example, human SELENOP has 10 residues, whereas the zebrafish protein has 17^5^. *Selenop* mRNA was down-regulated significantly in female *Sbp2* βCKO murine islets; however, expression in male mice was unchanged. Interestingly, Selenop protein levels did not differ in the *Sbp2* βCKO mice in either male or female mice, indicating a role for other post-translational mechanisms of regulation. Curiously, despite preservation of Selenop protein levels, gene expression of the Selenop receptor *Lrp8* was robustly increased in both sexes, while *Lrp1* expression was reduced selectively in female *Sbp2* βCKO mice. The implications of these findings with regard to intracellular selenium sensing and transport require further investigation.

Thyroid hormones play significant roles in β-cell development and function^40^, and tissue deiodinases regulate the activity of thyroid hormone signaling by locally modulating the levels of the active thyroid hormone T3 versus inactive metabolites. While Dio1 and Dio2 (deiodinase 1 and 2, respectively) primarily convert T4 to T3, Dio3 inactivates both T4 and T3^40^. Only *Dio1* was expressed in murine islets in our model. In contrast, Medina et al. previously reported high expression of *Dio3* in male murine β-cells with a mixed genetic background (129/Sv/C57BL/6)^41^. Interestingly, despite *Dio1* mRNA being downregulated in both male and female *Sbp2* βCKO mice, protein expression was found to be unchanged.

The thioredoxin reductases are another group of proteins important in islet biology^42^. Thiroredoxin reductases are integral components of the thioredoxin system in which thioredoxins catalyze the reduction of disulfide bonds in target proteins, with oxidized thioredoxin in turn being reversibly reduced by thioredoxin reductases^42^. Thioredoxins play significant roles in antioxidant defense as they contribute to the disulfide reductions in peroxiredoxins that are involved in the reduction of hydrogen peroxide and peroxides^43^. Thioredoxin reductases 2 and 3 (*Txnrd2* and *Txnrd3*, respectively) were the least expressed selenoprotein mRNAs in both male and female murine islets. Both *Txnrd2* and *Txnrd3* mRNA were downregulated in female *Sbp2* βCKO islets but not in male islets. Protein levels of *Txnrd2* were not influenced by *Sbp2* deletion.

While the present studies provide new insights into the selenoprotein complement of a critical metabolic tissue, these studies have several limitations. β-cell-specific deletion of *Sbp2* in murine islets affects only 60–80% of islets cells^25^. Because gene transcription was assessed in islets, the apparent impact of *Sbp2* gene deletion may have been diluted by the presence of non-β-cells in the tissue, which we might expect to have their own unique selenoprotein transcript expression profiles, distributions, and NMD susceptibilities. Further study of the role of SBP2 using purified β-cells or β-cell lines may clarify its role in selenoprotein transcription and translation. In addition, studies in α-cells may clarify potential compensatory changes in selenoprotein expression in islet tissue. Stability of protein expression despite *Sbp2* deletion and reduced mRNA levels points to a role for additional mechanisms that may operate post-translationally, and these require clarification. These mechanisms may contribute to the selenoprotein expression hierarchy in islets under normal conditions. Additional research is required to show how selenoprotein mRNA expression or protein levels change in pathological states such as diabetes and metabolic dysfunction. Further work is also required to interrogate the mechanisms responsible for the observed sex-specific effects on mRNA expression. Importantly, our studies employed Mip-Cre-ERT mice in which pancreatic β**-**cell-specific Cre expression is driven by the mouse *Ins*1 promoter; other β-cell-targeting Cre lines, such as those driven by the rat insulin promoter, are available and have been used to interrogate the impact of disrupting various aspects of selenoprotein biology on metabolic function^44,45^. Comparative application of these models and approaches may further illuminate the role of the selenoproteome in regulating metabolic health. We have limited our discussion to just *Gpx3*, *Selenop*, *Dio1*, and *Txnrd2* as these are the best studied selenoproteins with regard to pancreatic β-cell function and metabolic physiology. Other selenoproteins may play significant roles as well. For example, while not an NMD-susceptible selenoprotein, SELENOS is a suggested therapeutic target for diabetes^46^. In our studies *Selenos* mRNA expression is unchanged by *Sbp2* deletion in pancreatic islets from both sexes. As a major regulator of selenoprotein mRNA synthesis and mRNA decay^47^, our data from pancreatic islets of Langerhans add to existing information with regard to the role of SBP2 in NMD and in establishing the hierarchy of selenoprotein expression. However, further work is required to precisely clarify the role of this important protein in selenoprotein synthesis and its relative contribution to mRNA stability versus protein translation given evidence of preserved Sec insertion into proteins in *Sbp2*-deficient cells^47^.

Our report is the first to examine the complete sex-specific selenoprotein transcript profiles in β-cells and the influence of *Sbp2* deletion on transcription. Given the essential role of β-cells in maintaining glucose homeostasis, further studies examining islets from models of diabetes or those subjected to other metabolic stressors will clarify the dynamics of selenoprotein expression and their role in maintaining β-cell function and overall metabolic health.

## Supplementary information

Supplementary Information
