# Supplementary material for: Selenocysteine insertion sequence binding protein 2 (Sbp2) in the sex-specific regulation of selenoprotein gene expression in mouse pancreatic islets
Source: Sci Rep. 2020 Oct 29;10:18568. doi: 10.1038/s41598-020-75595-4 (PMC7596060; doi:10.1038/s41598-020-75595-4)
Supplement: Supplementary file 1 — Supplementary Information [file 41598_2020_75595_MOESM1_ESM.pdf]

Supplementary Figures

Title: Selenocysteine Insertion Sequence Binding Protein 2 (SBP2) in the Sex Specific Regulation of Selenoprotein Gene Expression in Mouse Pancreatic Islets

Authors: Chellan, B; Zhao, L; Landeche, M; Carmean, CM; Dumitrescu, AM; and Sargis, RM

Fig. 1c:

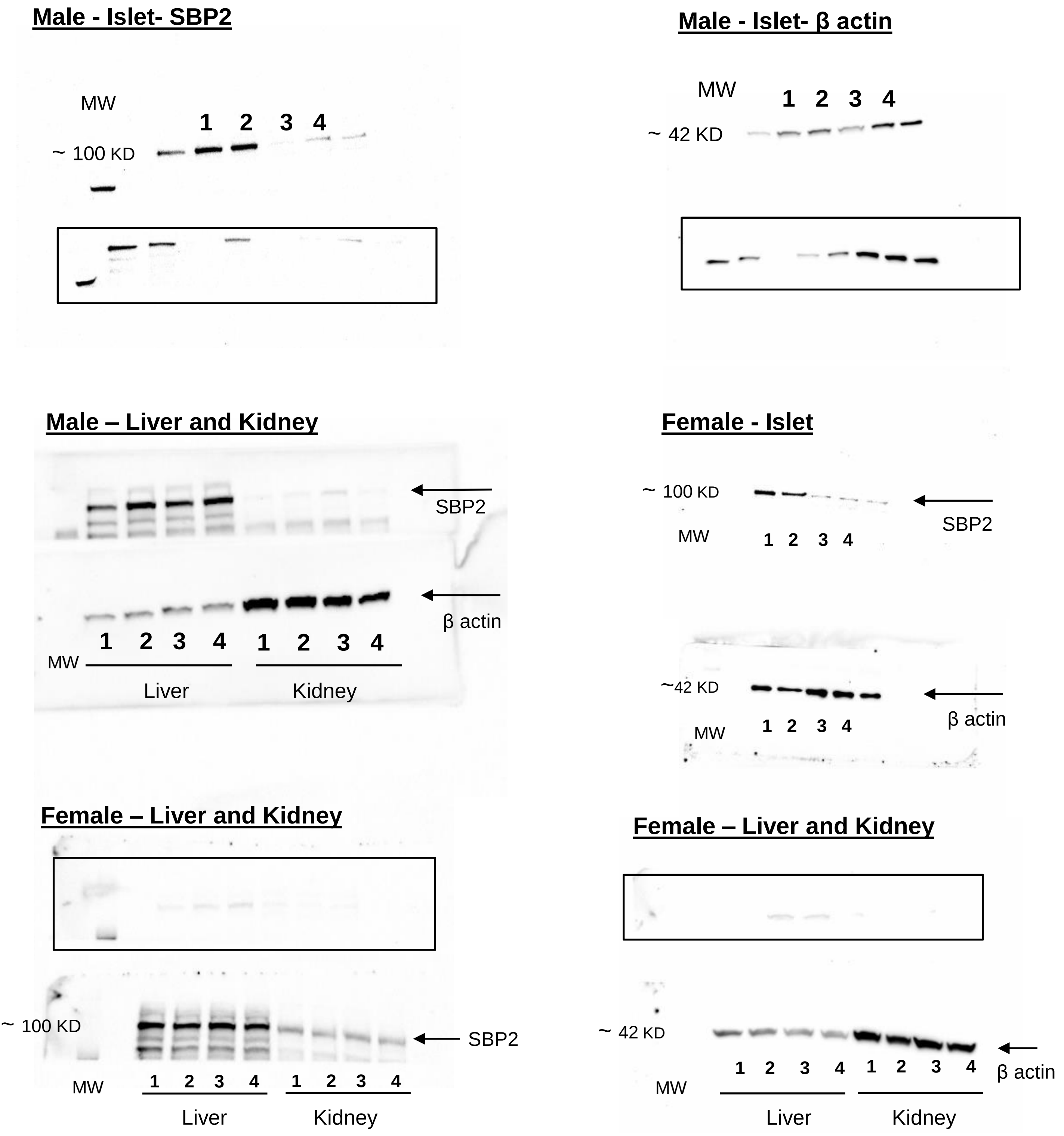

Whole Immunoblot showing SBP2 and β actin detection in male and female mouse tissues as shown in Fig.1c in main text; lanes 1 and 2 are from control mice (*Sbp2* fl/fl), and lanes 3 and 4 are from *Spb2* βCKO. The blots inset in box are unrelated, separate blots, photographed along with the SBP2 & β actin blot. MW- marker lane

Fig.3c:

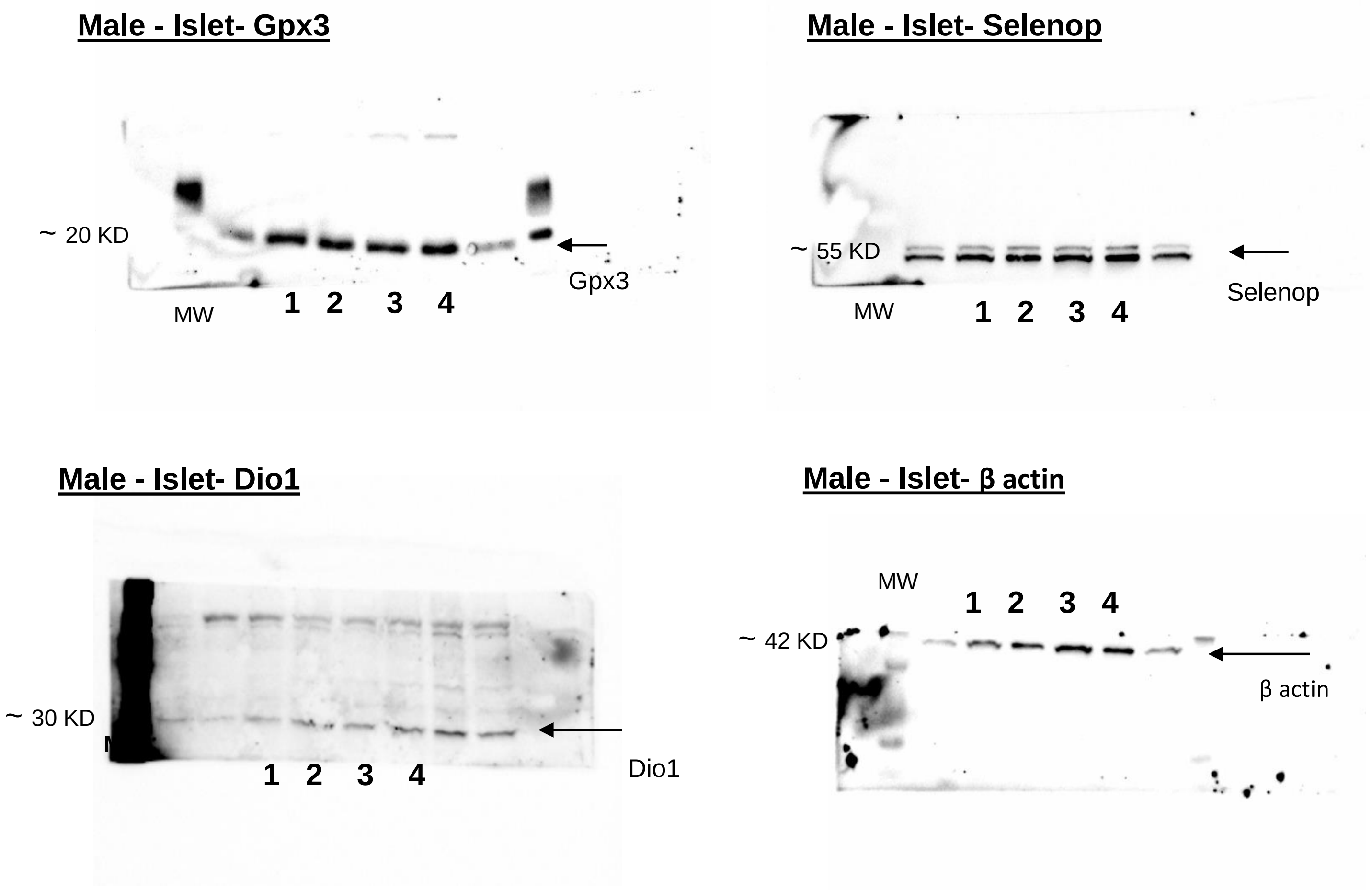

Whole Immunoblot showing Gpx3, Selenop, Dio1 and β actin detection in male mouse islets as shown in Fig.3c in main text; lanes 1 and 2 are from control mice (*Sbp2* fl/fl), and lanes 3 and 4 are from *Spb2* βCKO. MW- marker lane

Fig.3d:

Female - Islet- Gpx3

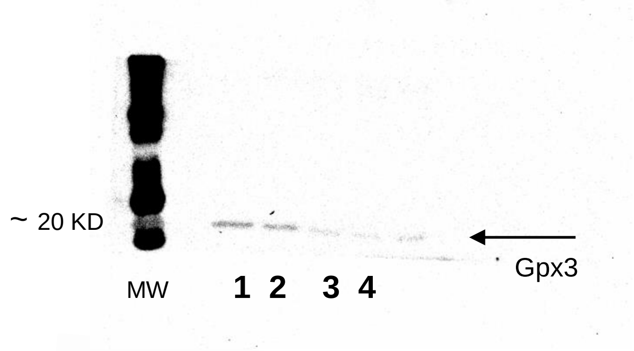

Female - Islet- Selenop

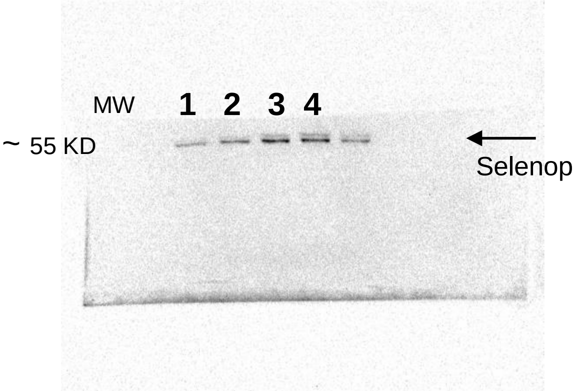

Female - Islet- Dio1

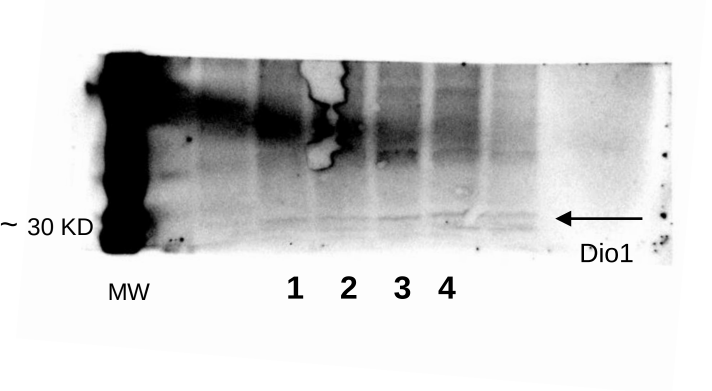

Female - Islet- Txnrd2

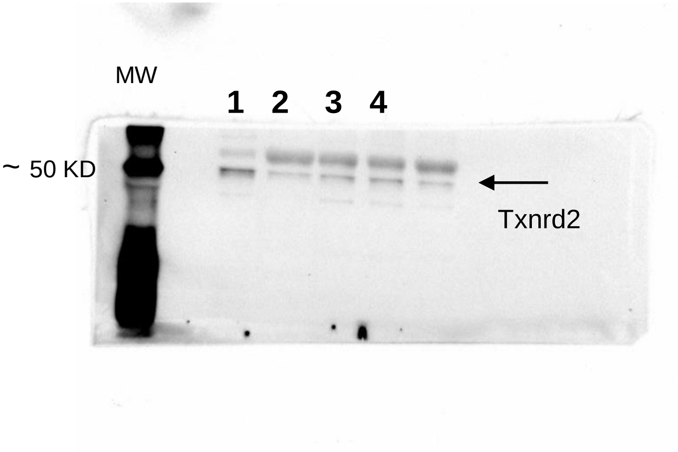

Female - Islet- β actin

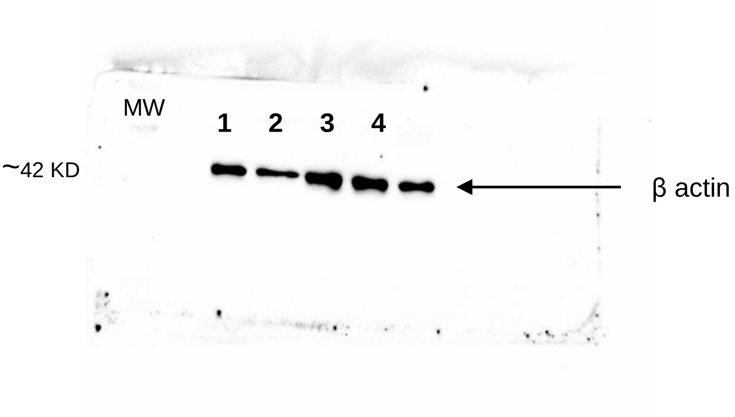

Whole Immunoblot showing Gpx3, Selenop, Dio1, Txnrd2 and β actin detection in Female mouse islets as shown in Fig.3d in main text; lanes 1 and 2 are from control mice (*Sbp2* fl/fl), and lanes 3 and 4 are from *Spb2* βCKO. MW- marker lane
